# Three types of scientific evidence to inform physical activity policy: results from a comparative scoping review

**DOI:** 10.1007/s00038-016-0807-y

**Published:** 2016-04-26

**Authors:** Alfred Rütten, Diana Schow, João Breda, Gauden Galea, Sonja Kahlmeier, Jean-Michel Oppert, Hidde van der Ploeg, Willem van Mechelen

**Affiliations:** Institute of Sport Science and Sport, Friedrich Alexander University, Erlangen, Germany; Division of Noncommunicable Diseases and Life-course, WHO Regional Office for Europe, Copenhagen, Denmark; Physical Activity and Health Unit, Epidemiology, Biostatistics, and Prevention Institute (EBPI), University of Zurich, Zurich, Switzerland; Department of Nutrition, University Pierre et Marie Curie – Paris 6, Pitie-Salpetriere hospital (AP-HP), Institute of Cardiometabolism and Nutrition (ICAN), Paris, France; Department of Public and Occupational Health and EMGO+ Institute, VU University Medical Center, Amsterdam, The Netherlands

**Keywords:** Physical activity, Policy, Health promotion, Scoping review, Evidence

## Abstract

**Objectives:**

This paper presents a typology of available evidence to inform physical activity policy. It aims to refine the distinction between three types of evidence relating to physical activity and to compare these types for the purpose of clarifying potential research gaps.

**Methods:**

A scoping review explored the extent, range and nature of three types of physical activity-related evidence available in reviews: (I) health outcomes/risk factors, (II) interventions and (III) policy-making. A six-step qualitative, iterative process with expert consultation guided data coding and analysis in EPPI Reviewer 4.

**Results:**

856 Type I reviews, 350 Type II reviews and 40 Type III reviews were identified. Type I reviews heavily focused on obesity issues (18 %). Reviews of a systematic nature were more prominent in the Type II (>50 %). Type III reviews tended to conflate research about policy intervention effectiveness and research about policymaking processes. The majority of reviews came from the United States, United Kingdom, Australia and Canada.

**Conclusions:**

Although evidence gaps exist regarding evidence Types I and II, the most prominent gap regards Type III, i.e. research pertaining to physical activity policymaking. The findings presented herein will be used to inform physical activity policy development and future research.

**Electronic supplementary material:**

The online version of this article (doi:10.1007/s00038-016-0807-y) contains supplementary material, which is available to authorized users.

## Introduction

The need for a scoping review on physical activity evidence types is two-fold: First, the World Health Organization (WHO) Regional Office for Europe and its Member States are implementing the first European Strategy on Physical Activity to support the reversal of trends in rates of certain non-communicable diseases and obesity (World Health Organization^ 2014^). They seek to ground the strategy on current scientific evidence. Second, in a WHO working group convened to inform the new strategy, experts agreed that the rapid increase of published research on the subject of physical activity necessitates a fresh look at the body of available evidence. Of special interest is the distinction between three types of evidence: Type I evidence pertains to physical activity and health, i.e. studies that link physical activity to risk factors or health outcomes. Type II evidence pertains to physical activity interventions, i.e. studies that link interventions to physical activity behavior. Type III evidence pertains to physical activity policy, i.e. studies that link policymaking to physical activity (cf. Brownson et al. 2009; Martin-Diener et al. 2014).

Some assumptions were made regarding evidence types before starting the scoping review. Policy-makers and researchers involved agreed that Type I and Type II evidence might be much more developed than Type III evidence. For example, the landmark review of the Physical Activity Advisory Committee (United States Department of Health and Human Services 2008) provided an extensive overview of Type I evidence. Likewise, Type II evidence has been broadly covered in previous publications e.g. Heath et al. (2012) and World Health Organization (2009). In contrast, very few attempts have been made to specifically establish the evidence-base for physical activity policy-making. Some approaches have developed good practice criteria based on literature reviews (Bull et al. 2014). Others completed content analyses of national-level policy documents (Daugbjerg et al. 2009; Vestmark et al. 2011) or focused on the use of evidence in physical activity policies (Aro et al. 2015).

To our knowledge no review systematically integrates and compares the literature available across the three different evidence types. A comparison of this kind would increase understanding of the extent, range and nature of the available evidence and the evidence gaps in physical activity research. It could also be used to ground policies on all types of current scientific evidence. Moreover, knowledge derived from the comparison might be useful to guide future research, support efforts of pivotal organizations (Martin et al. 2006) and encourage innovative funding schemes.

We first explain our conceptual approach. Second, we give an overview of the scoping review methodology. Third, we present comparative results of the three evidence types regarding (a) number of reviews found, (b) kinds of reviews, (c) first author country affiliation, (d) dates of publications, and (e) most frequent and/or trending topics. Fourth, in the discussion and concluding remarks we relate results to previous work, discuss limitations of the study and indicate future research needs.

### Conceptual approach

A key aim of this paper is to refine the distinction between three types of evidence relating to physical activity. A second aim is to compare evidence types for the purpose of identifying potential research gaps. Type I evidence focuses on the link between physical activity and health status, i.e. the type, amount and intensity of physical activity and its effects on different health outcomes. For example, this type of evidence might demonstrate how physical activity reduces the risk of diabetes. Type II evidence links interventions with physical activity, i.e. the type of intervention, setting and/or environment that influences physical activity. For example, this type of evidence might demonstrate how settings-based interventions support increased physical activity. Effective environmental and policy interventions to promote physical activity also belong to Type II evidence. Type III evidence focuses on the effects of policy-making on physical activity interventions, i.e. the policy agendas, structures, funding and processes that affect development, implementation or adaptation of physical activity interventions. For example, this type of evidence may demonstrate how cross-sectoral approaches to policy-making help position physical activity promotion on the agendas of different policy sectors and policy levels.

Regarding Type II and Type III evidence, policy plays a role in both. Policy is involved in Type II evidence as a part of interventions to increase physical activity. Policy-involved interventions, however, result from the dynamics of policy-making processes (Type III evidence). For example, tax incentives that encourage active transportation may result in more active lifestyles in the general population. These incentives, as part of interventions, can be deemed Type II evidence. The policy-making process of reaching consensus on the incentives and examining the political environment can be deemed Type III evidence.

The distinction between Type I and Type II evidence is widely addressed in the literature. Making the distinction between Type II and III evidence is more innovative. Doing so may help overcome conceptual hurdles in previously published literature, which often conflates “policy and environmental approaches” or deals with policy approaches as a type of (physical activity) intervention (Brownson et al. 2006; Matson-Koffman et al. 2005; Rychetnik et al. 2002; Sallis et al. 1998). We propose to better distinguish research about policy interventions (including specific programs and projects) from policy-process oriented research that may more often be explored by the disciplines of social or political science. We recognize that some reviews may contain evidence relevant to more than one evidence type. This scoping review was designed to identify the main emphasis in each of the review articles that were analyzed to ascertain how the different types of evidence are being prioritized in the literature. This distinction is being made for conceptual, research-driven, and policy-driven reasons.

First, a conceptualization of the evidence of physical activity policy-making is needed. It will lend to a better understanding, for example, of how policy-making structures and processes in different countries influence common factors associated with effective physical activity interventions. This conceptual perspective builds on previous models and frameworks that connected policy issues with physical activity. For example, Sallis et al.’s (2006) ecological model for active living refers to a distinct category of the policy environment. Likewise, Schmid et al.’s (2006) framework for physical activity policy research underlines the need for research on policy agenda setting (“the determinants of establishing policy”, pS22) and policy processes (“process of developing and implementing policies” pS22). Most recently, Rütten et al.’s (2013) theory-driven model focuses on the interplay of policy, the environment, and physical activity behavior.

Second, there is an emerging body of research outside the physical activity context, but highly relevant to it, with a special focus on policy agenda setting, policy structures and processes in public health (Commission on Social Determinants of Health 2008). This research points to distinct evidence types regarding policy-making. For example, Lin et al. (2012) synthesized the evidence on “how governance structures can trigger governance action to support Health in All Policies”. Similar to our focus on a distinct evidence type related to physical activity policy-making, this evidence synthesis “was developed to advance knowledge on how to effect” policies (in this case Health in All Policies) through policy-making (here: “through intersectoral governance”) (Lin et al. 2012, p23). Moreover, there are several recently published reviews that focus on the evidence of intersectoral policy-making on social determinants of health and on health equity (e.g. Chircop et al. 2014; Ndumbe-Eyoh and Moffatt 2013; Rantala et al. 2014; Shankardass et al. 2012).

Third, there is a need for specific recommendations based on Type III evidence for physical activity policy development and implementation both at international and national levels. One example is the aforementioned WHO European Physical Activity Strategy. Inclusion of detailed recommendations about evidence-based strategies regarding agenda setting and other policy processes stands to strengthen and complement recommendations associated with Type I and Type II evidence. Another example is related to the European Commission’s efforts to implement the EU Physical Activity Guidelines (2008) as a framework for policy development (The Council of the European Union 2013, C 354/2).

Finally, when establishing a conceptual approach to reviewing three types of evidence similar perspectives were sought within public health literature. Comparable distinctions of evidence types have been made by Brownson et al. (2009) and Martin-Diener et al. (2014). Type III evidence in Brownson et al. (2009), however, does not specifically relate to policy (which is essential for our approach). Instead, it relates to “context” in a much broader sense. Their Type III pertains to evidence “needed to adapt and implement an evidence-based intervention” (2009, 179). In their framework Type III “political and economic” evidence describes just one kind of context variable at a particular level, which is distinguishable from other Type III context variables at other levels (e.g. “individual, interpersonal, organizational, and sociocultural). Martin-Diener et al. (2014, 8) integrated the typology of Brownson et al. (2009) in their “HEPA Europe Framework”. They relate Type III evidence to a much broader type of information on “How can be done what should be done?” They explicitly mention “policy process” as a category related to this evidence type but do not further conceptualize in this paper what that means related to their broad-ranging question quoted above.

### Research question

The research question was clarified in an August 2014 sub-committee meeting of the WHO working group on a European Physical Activity Strategy. It was designed to foster three different, yet comparable, lines of systematic inquiry regarding the evidence types, with a pre-established recognition that Type III evidence is increasingly relevant: The question was also designed to ensure that research was gathered from studies across the lifespan.

What are the extent, range and nature of scientific evidence that exists relating to physical activity in three different areas?

- Type I—evidence that links physical activity to health outcomes.
- Type II—evidence that links interventions to physical activity behavior.
- Type III—evidence that links policy-making to interventions.

## Methods

Scoping reviews systematically synthesize knowledge (Grimshaw 2010) to ascertain the extent, range and nature of research in a particular area of inquiry (Arksey and O’Malley 2005). They usually involve iterative, qualitative approaches to analyzing, describing, mapping and reinterpreting available research (Levac et al. 2010). This is useful for gaining a better understanding of gaps in evidence, pivotal concepts, perspectives and trends in research. It is also useful for determining whether further exploration and analysis of the literature might be prudent (Armstrong et al. 2011).

This scoping review was conducted by a research sub-committee of the working group from the WHO European Strategy on Physical Activity for the purpose of overcoming the conceptual vagueness and blurry distinctions between the three types of evidence mentioned above, and to advance the knowledge base regarding the place of policy in the increasingly relevant implementation of physical activity interventions to combat social inequalities and non-communicable diseases.

**Fig. 1:**
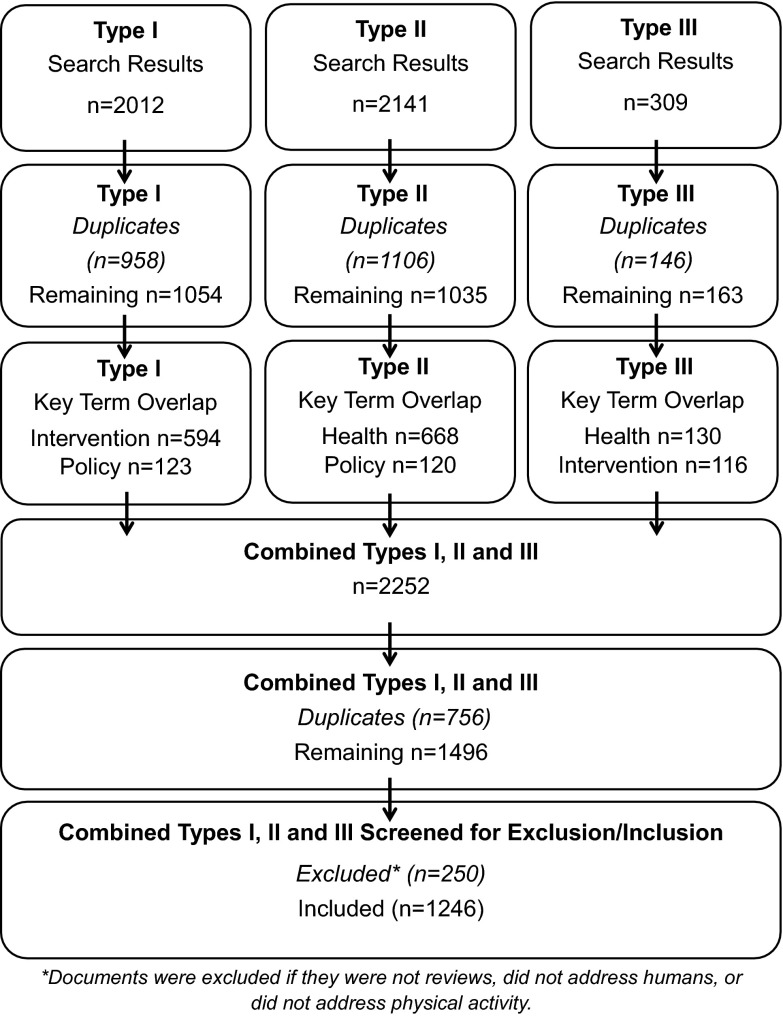
Scoping review search process

In July 2014 the research sub-committee began a six-step scoping review process, adhering to the methodological framework set forth in Arksey and O’Malley (2005) and expanded by Levac et al. (2010): (1) clarify the research question, (2) identify relevant studies, (3) select studies, (4) chart and describe studies and data, (5) summarize and report results, (6) engage in consultation process with sub-committee members and key stakeholders. Sub-committee members represented a multi-disciplinary team of academics, policymakers and practitioners. Library scientists at Friedrich Alexander University, Erlangen-Nuremberg were consulted throughout in reference to the selection of key search terms, databases and pilot search processes. As part of Step 6 all scoping review activities involved ongoing consultation (e.g. in-person, telephone and Skype meetings) with stakeholders on the research sub-committee. Meetings were held at the outset of the project and at the key intervals of data collection and analysis addressed in Fig. 1 below. It was at these meetings that it was determined how to proceed with data analysis and reporting.

In this section we focus on Arksey and O’Malley (2005) steps 1, 2 and 3. Steps 4 and 5 are addressed in the following section, where results are charted and summarized. Step 6 (consultation with relevant stakeholders) is addressed throughout the discussion.

### Identifying relevant studies

Relevant studies were identified between September 2014 and November 2014. Pilot searches took place between October 8 and 31, 2014. Final searches took place between November 1 and 15, 2014. Selection and analysis of relevant studies took place between December 2014 and May 2015. Key search terms were selected based on results from more than 200 pilot searches along with a backwards keyword search (Levy and Ellis 2006). The backwards keyword search was done using sets of pre-identified core documents compiled for each evidence type. Final search terms were considered in relation to grammatical variation, i.e. singular and plural form, tense, etc. They were selected and defined based on each evidence type:

- Type I combined variations of the terms “health, physical activity, evidence, effect and review”.
- Type II combined variations of the terms “intervention, physical activity, evidence, effect and review”.
- Type III combined variations of the terms “policy, physical activity, evidence, effect and review”.

Titles and abstracts in PubMed, Scopus, SportDiscus, PsycInfo, ERIC (Education Resource Information Center) and IBSS (International Bibliography of the Social Sciences) were searched independently by two researchers and in consultation with an institutional librarian. Searching incorporated Boolean operators, advanced search features and grammatical variations of search terms. While the type and number of search terms could easily be expanded in any direction, especially in relation to synonyms for physical activity (e.g. exercise), doing so would make the comparison of results across evidence types inconsistent. This review, however, is intended to be the first of a series of reviews that will build upon results in a step-wise process. The next round of reviews may tailor new combinations of keywords based upon what was learned from this review.

### Inclusion criteria and exclusion criteria

At the outset, it was recognized that there might exist a vast amount of research in one area and a limited amount of research in another. Therefore, it was determined that prior to exploring single studies, the literature would be searched for various types of reviews. A variety of kinds of reviews was purposefully sought (e.g. systematic reviews, narrative reviews, non-systematic reviews), which fostered openness to identifying evidence produced from different disciplinary sources (e.g. public health, clinical medicine, social science, political science). All inclusion/exclusion criteria for the initial search, which sought to compare across the three different types of evidence, was the same. Any document type in English, French, German or Spanish was considered. Any study focusing on humans that addressed physical activity and was discussed by the authors in relation to effectiveness of evidence was considered, regardless of outcome, type of intervention, target group or exposure.

Documents were excluded if they did not address humans, made no connection to physical activity or were not considered to be an evidence review. For example, while some documents may have been retrieved that contained the word “review”, they may have, in fact, been a single study. Those remaining were included as part of conceptual mapping and comparative analysis.

### Selecting relevant studies

Relevant studies were selected and analyzed between December 2014 and May 2015. A team of three researchers checked for duplicate references using EndNote plus a manual duplicate search. Once duplicates (Type I *n* = 958, Type II *n* = 1106, Type III *n* = 146) were removed, the research team conducted a preliminary screening of the remaining documents (Type I *n* = 1054, Type II *n* = 1035 Type III *n* = 163) in each category. After the preliminary screening it was found that the titles and abstracts contained a significant overlap in relation to the different evidence types (i.e. health, intervention and policy) (see Fig. 1 for overlap details). Because of this overlap, it was decided to combine the documents (*n* = 2252), eliminate duplicates (*n* = 756) in a second step and then conduct a screening and sorting process on the remaining documents (*n* = 1496).

References were then imported into EPPI Reviewer 4 where a team of researchers independently assessed a sample of the documents. A final screening of all references eliminated false positives (*n* = 250) (e.g. those that were not reviews, did not address physical activity, or did not relate to humans). All documents not identified as false positives were deemed relevant and selected for qualitative comparison (*n* = 1246).

## Results

According to the conceptual approach described above, 856 reviews were classified as Type I, 350 as Type II, and 40 as Type III. The clear difference in totals confirms the initial hypothesis of the working group that Type III evidence is less developed and less differentiated in terms of conceptual variables that might advance the field of physical activity policy-making research.

For more detailed description, the results were sorted into cascading sub-categories: (1) kind of review, (2) country of first author’s institution, (3) date of publication, and (4) emergent themes.

**Fig. 2:**
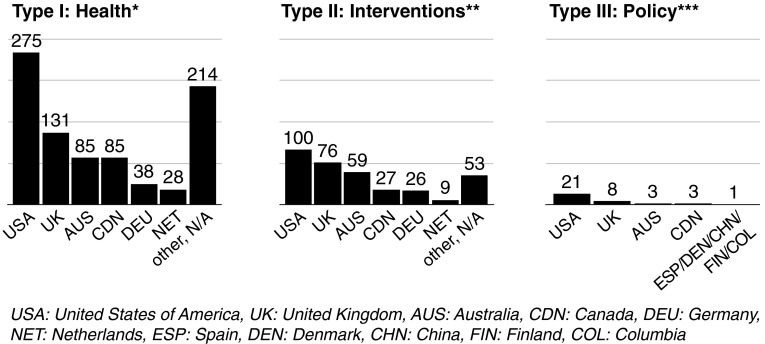
First author institutional affiliation by evidence type and country

There are a variety of kinds of reviews to consider when ascertaining the extent, range and nature of research that originates from different disciplines (Coughlan et al. 2013). In relation to physical activity, systematic reviews are often used to synthesize results from quantitative studies such as clinical trials and appraise effectiveness of interventions (Grant and Booth 2009). They tend to adhere to guidelines such as those put forth by Cochrane (Higgins and Green 2011). Meta-analyses combine results from systematic reviews and present additional analyses. In contrast, qualitative reviews, concept analyses and narrative reviews do things such as clarify conceptual approaches to interventions, describe existing qualitative evidence and effectiveness of interventions and critique claims of effectiveness of interventions. Different types of reviews contribute to the evidence-base in meaningful ways. Systematic reviews and meta-analyses are most prominent regarding Type II evidence (more than 50 %) and represent almost 40 % of selected reviews of Type I evidence (see Additional File 1, Table 1). In contrast, these approaches only represent 20 % of the Type III reviews. The bulk of research in each evidence category was generated in the United States, United Kingdom, Australia and Canada. Two thirds of Type I reviews, three-quarters of Type II reviews and almost 90 % of Type III reviews had first author institutions from these four countries (see Fig. 2).

**Fig. 3:**
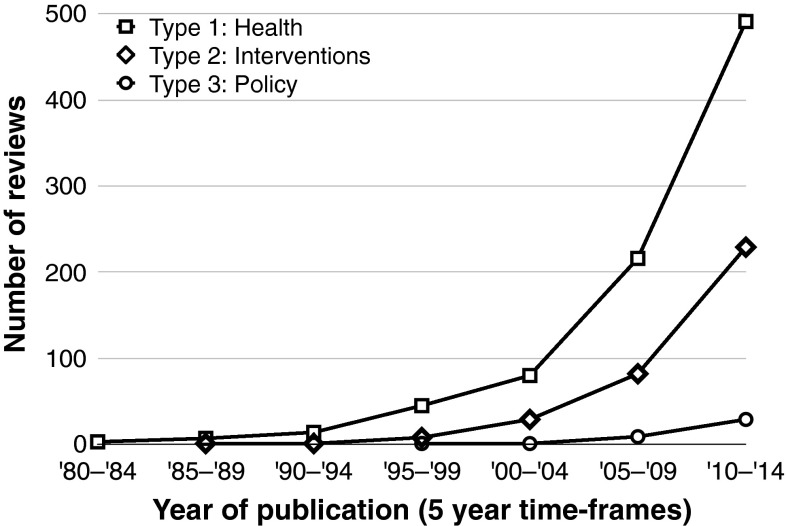
Distribution of studies according to evidence type and year of publication

Fifty-seven percent of Type I reviews were published between 2010 and 2014 and twenty-five percent were published between 2005 and 2009. The remaining eighteen percent were published between 1980 and 2004. Sixty-five percent of Type II reviews were published between 2010 and 2014 and twenty-three percent were published between 2005 and 2009. The remaining eleven percent were published between 1980 and 2004.^1^ Seventy-three percent of Type III reviews were published between 2010 and 2014 and twenty-three percent were published between 2005 and 2009. The remaining four percent were published between 1980 and 2004 (Fig. 3).

**Fig. 4:**
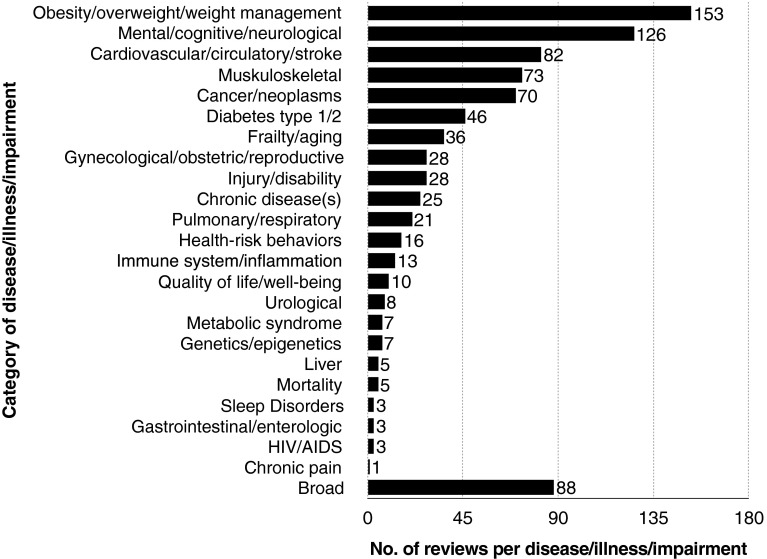
Classifications of type I (health) reviews relating to disease/illness/impairment

Eighteen percent of Type I reviews (Fig. 4) have a primary emphasis on obesity/overweight/weight management. Fifteen percent had a primary emphasis on mental/cognitive/neurological issues. Following these in descending order the combination of: (1) cardiovascular/circulatory/stroke issues (2) musculoskeletal issues (3) cancer/neoplasms and 4) diabetes types 1 and 2 represent thirty-two percent of reviews. Ten percent of reviews were categorized as “broad” because they addressed a variety of diseases/illnesses and/or impairments.

**Fig. 5:**
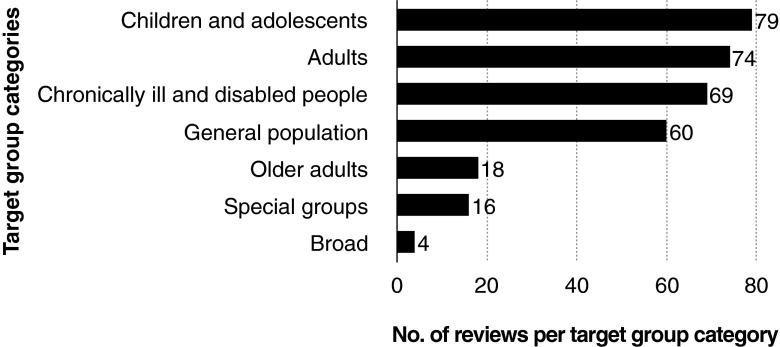
Classifications of type II (intervention) reviews based on target groups

Regarding Type II (Fig. 5), the most prevalent target groups that reviews focused on were children and adolescents (23 %), followed closely by adults (21 %) and chronically ill or disabled (20 %). Older adults (5 %) and special groups (5 %) were less often addressed. Ten percent of reviews were classified as “broad” because they included studies relating to more than one target group or were not focused on addressing target groups.

The most prevalent setting in Type II was health care (31 %, see Additional File 1, Fig. 1). This was followed by community and city (13 %), school, after school (i.e. the after school period during the day), university (11 %), worksite (6 %) and home (3 %), respectively. Thirty-five percent of reviews were categorized as broad if they addressed multiple settings or were not focused on settings. The most prevalent intervention type in Type II reviews was counseling/education/referral (24 %, see Additional File 1, Fig. 2). This was followed by technology and computer-based (10 %), exercise and training (9 %), information-based (8 %), environmental (7 %), social and organizational (7 %), community-based (3 %) and mass media (1 %). Thirty percent of reviews were categorized as “broad” if they addressed multiple intervention types or were not focused on interventions.

**Fig. 6:**
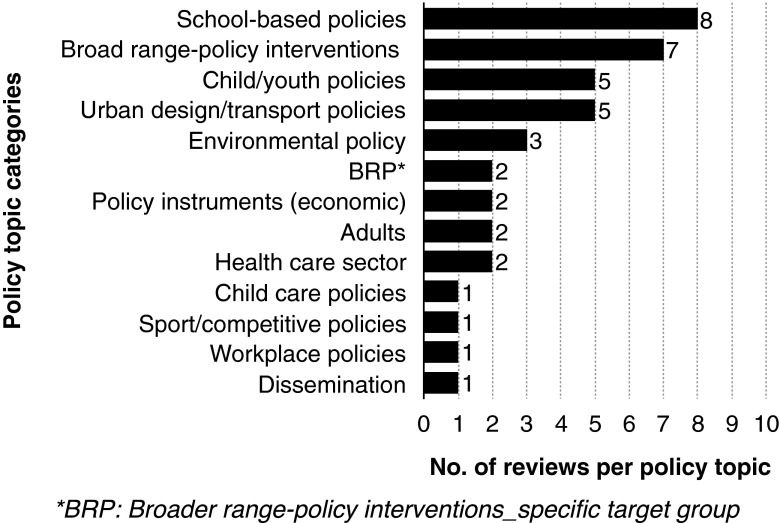
Classification of type III (policy) reviews

The majority of Type III policy reviews focused on school-based polices (20 %) (Fig. 6). This was followed by broad-range policy interventions (i.e. those that reach across more than one topic) (17.5 %), child/youth policies (12.5 %), urban design/transport policies (12.5 %) and environmental policy (7.5 %). Broader range policy interventions that address a specific target group, policy instruments, adults and health care sector each represented 5 % of reviews. Child care policies, sport/competitive policies, workplace policies and dissemination represented 2.5 % of reviews.

## Discussion

This discussion addresses how the results of this scoping review achieve the aims of clarifying the distinction between evidence types and helping understand where potential research gaps can be filled. It also provides insights into evidence-based policy-making in the areas of health and health promotion in several ways.

First, the outcomes confirmed the hypothesis that evidence for the three types of physical activity research differs in terms of quantity and characteristics of reviews available in scientific journals. For example, the number of reviews on health effects of physical activity (Type I) was more than twenty times higher than those on physical activity policy-making (Type III). The kind of reviews ranked highest in the hierarchy (Higgins and Green 2011), e.g. meta-analyses and systematic reviews, were most prominent in research on physical activity interventions (Type II) and were least found in policy-related reviews. Some of this difference may be attributed to the appropriateness of study relevant to each evidence type. Type III evidence may be more appropriate for analysis in reviews that are not deemed systematic. Even so, the number of Type III reviews found was much lower than Type I and Type II reviews. These outcomes mirror the conclusion from Breton and de Leeuw’s (2008) systematic review that policy research in health promotion is still in its infancy. They also demonstrate that previous calls from researchers and public health organizations (e.g. Schmid et al. 2006) to give higher priority to policy-related research on physical activity are still very relevant today.

Second, although this scoping review was not limited to English language publications, most results show a first author affiliation for the United States, United Kingdom, Australia, or Canada. This implies a need to: (1) extend the scoping review to other than English languages/data bases to determine the availability of evidence from non-English language sources, (2) consider placing more emphasis on policy-related physical activity research in low and middle income countries (e.g. revisit the distribution of funding support, increase access to technological resources and implement capacity development measures for researchers and their institutions) (see Kohl et al. 2012) and (3) draw attention to this dynamic when conducting or reviewing research that accompanies current policy strategies in Europe such as implementation of the WHO Physical Activity Recommendations, EU Physical Activity Guidelines (EU Working Group “Sport and Health” 2008) and development of the new WHO European Physical Activity Strategy.

Third, the results demonstrate the current dynamic of physical activity research. It started to grow in the mid-1990s but developed more rapidly in the mid-2000s. The initial development may reflect the research impact of landmark scientific publications and policy documents, e.g. the US shift in recommendations towards the concept of health-enhancing physical activity (Pate et al. 1995), the Surgeon General’s Report on Physical Activity and Health (United States Department of Health and Human Services 2008), and the WHO Global Strategy on Diet and Physical Activity (World Health Organization 2004). More than half of the reviews in each evidence type were published in the last five years. While the absolute numbers of reviews found for this time period are the highest in Type I (about 500), compared to Type II (230) and Type III (about 30), the relative increase is highest in the policy related category where almost three quarters of the reviews were published since 2010. This may reflect an increasing emphasis on physical activity policy research by important public health organizations (see Schmid et al. 2006).

Fourth, the majority of Type I reviews primarily focused on obesity/overweight and weight management (e.g. Astrup 1999; Bonfioli et al. 2012; McPherson et al. 2014). These reviews merit further consideration in relation to the link between physical activity, weight management and different types of health outcomes. Further analysis might, for example, identify what types of interventions were used in the studies that were reviewed to promote obesity reduction. Thirteen of the Type I reviews reflect a potential growing interest in inflammation/immunity, as reflected in the literature, (Harvey et al. 2011; Lee and Pratley 2005), and how physical activity may play a key role (Packer et al. 2010, Romeo et al. 2010). Eleven percent of Type I reviews contained the word “sedentary” in either the title or abstract, reflecting increasing trends in relation to health outcomes and sedentary behavior.

Fifth, Type II evidence reviews showed an emphasis on health care in terms of settings (31 %) (e.g. Hinrichs and Brach 2012; Vancampfort et al. 2012) and young people in terms of target groups (23 %) (e.g. Chaput et al. 2013; Floriani and Kennedy 2007). In terms of intervention types, counseling/education/referral reviews (e.g. Cramp et al. 2013) are highly represented (24 %) as traditional forms of physical activity and health promotion. The presence of technology/computer focused intervention types (10 %) (e.g. Hamel et al. 2011) may signal their increasing role in physical activity promotion. Fourteen percent of Type II reviews contained the word “sedentary” in either the title or abstract, also reflecting trends in relation to physical activity interventions and sedentary behavior.

Sixth, as anticipated prior to this review, the results in the Type III category tend to address policy effectiveness (e.g. de Nazelle et al. 2011; Heath et al. 2006; Kumanyika et al. 2014), conflate policy with environmental approaches (e.g. Barton 2009; Sallis et al. 1998; Shill et al. 2012) and focus more on research as a method to influence creation of policy rather than on research as a method to assess policy-making processes, with the caveat that some studies do touch on this aspect (e.g. Patrick et al. 2009; Wiseman 2010). It may be that fewer Type III reviews were retrieved because conducting Type III studies would logically occur after Type I and Type II studies on a particular subject have already been completed, putting such studies further into the future and possibly out of reach of practical time constraints. Additionally, studies differ in their levels of complexity, and therefore feasibility, making Type III studies more challenging to implement in certain social, economic or political environments. It is the intention of the WHO European Strategy for Physical Activity research sub-committee to continue scoping the Type III evidence regarding physical activity and policy-making.

### Limitations

There are limitations to this study. First, the heuristic of the three types of evidence was limited. An overlap of evidence types exists within some of the reviews. For example, reviews that focused on policy and environmental interventions also included evidence about policy-making processes. To address this issue reviews were categorized by the evidence type that was most emphasized. Second, the three types of evidence explored were not intended to capture other important physical activity evidence review types, such as physical activity determinants (e.g. Bauman et al. 2012). This review is a first step in looking to new avenues of extracting and selecting what is “relevant” from the large quantity of literature that is retrieved through electronic resources used for this type of qualitative review.

### Conclusions

The distinction between three types of evidence serves as a useful heuristic to review the scope, dynamic and diversity of physical activity research in public health. This review demonstrates that this field is rapidly developing. Nonetheless, the production of Type III evidence is still in its infancy. It has been indicated that certain policy influences (e.g. Surgeon General Report (US DHHS 1996), WHO Global Strategy (WHO 2004) may have supported the production of Type I and Type II evidence in the past. Likewise, on-going policy initiatives such as the new WHO Physical Activity Strategy for the European Region could support the production of Type III evidence by emphasizing respective policy-related research. Continued analysis of documents within this scoping review is planned. It will focus on key studies that can inform recommendations regarding how to increase the quantity and the quality of Type III evidence with an emphasis on integrating stronger theoretical foundations and improving policy-process research methodology for the area of physical activity. While not within the purview of this review, it is important to mention that in the last decade sedentary behavior has emerged as a health risk, independent of physical activity. Further research will consider this topic. It will also seek to clarify the distinction between Type II evidence, i.e. what works in physical activity promotion, and Type III evidence, i.e. what works in policy-making for the purpose of developing concepts and providing an appropriate framework to guide future implementation and research.

## Electronic supplementary material

Below is the link to the electronic supplementary material.

Supplementary material 1 (PDF 175 kb)

## Footnotes

1 Percentages for Type II do not add up to 100 % due to rounding issues.

## References

[CR2] Arksey H, O’Malley L (2005). Scoping studies: towards a methodological framework. Int J Soc Res Methodol.

[CR3] Armstrong R, Hall BJ, Doyle J, Waters E (2011). ‘Scoping the scope’ of a Cochrane review. J Public Health.

[CR4] Aro AR, Bertram M, Hämäläinen RM (2015). Integrating research evidence and physical activity policy making—REPOPA project. Health Promot Int.

[CR5] Astrup A (1999). Physical activity and weight gain and fat distribution changes with menopause: current evidence and research issues. Med Sci Sports Exerc.

[CR6] Barton H (2009). Land use planning and health and well-being. Land Use Policy.

[CR7] Bauman AE, Reis RS, Sallis JF, Wells JC, Loos RJ, Martin BW, Lancet Physical Activity Series Working Group (2012) Correlates of physical activity: why are some people physically active and others not? Lancet. doi:10.1016/S0140-6736(12)60735-1 10.1016/S0140-6736(12)60735-1 22818938

[CR8] Bonfioli E, Berti L, Goss C, Muraro F, Burti L (2012). Health promotion lifestyle interventions for weight management in psychosis: a systematic review and meta-analysis of randomised controlled trials. BMC Psychiatry.

[CR9] Breton E, deLeeuw E (2008). Theories of the policy process in health promotion. Health Promot Int.

[CR10] Brownson RC, Haire-Joshu D, Luke DA (2006). Shaping the context of health: a review of environmental and policy approaches in the prevention of chronic diseases. Annu Rev Public Health.

[CR11] Brownson RC, Fielding JE, Maylahn CM (2009). Evidence-based public health: a fundamental concept for public health practice. Annu Rev Public Health.

[CR12] Bull F, Milton K, Kahlmeier S (2014). Turning the tide: national policy approaches to increasing physical activity in seven European countries. Br J Sports Med.

[CR13] Chaput JP, LeBlanc AG, McFarlane A (2013). Active healthy kids Canada’s position on active video games for children and youth. Paediatr Child Health.

[CR14] Chircop A, Bassett R, Taylor E (2014). Evidence on how to practice intersectoral collaboration for health equity: a scoping review. Crit Public Health.

[CR15] Commission on Social Determinants of Health (2008). Closing the gap in a generation: health equity through action on the social determinants of health. Final Report of the Commission on Social Determinants of Health.

[CR16] Coughlan M, Ryan F, Cronin P (2013). Doing a literature review in nursing, health and social care.

[CR17] Cramp F, Berry J, Gardiner M, Smith F, Stephens D (2013). Health behaviour change interventions for the promotion of physical activity in rheumatoid arthritis: a systematic review. Musculoskelet Care.

[CR18] Daugbjerg SB, Kahlmeier S, Racioppi F, Martin-Diener E, Martin B, Oja P, Bull F (2009). Promotion of physical activity in the European region: content analysis of 27 national policy documents. J Phys Act Health.

[CR19] de Nazelle A, Nieuwenhuijsen MJ, Antó JM (2011). Improving health through policies that promote active travel: a review of evidence to support integrated health impact assessment. Environ Int.

[CR1] EU Working Group “Sport and Health” (2008) EU physical activity guidelines. Recommended policy actions in support of health-enhancing physical activity. Brussels

[CR20] Floriani V, Kennedy C (2007). Promotion of physical activity in primary care for obesity treatment/prevention in children. Curr Opin Pediatr.

[CR21] Grant MJ, Booth A (2009). A typology of reviews: an analysis of 14 review types and associated methodologies [Review]. Health Info Libr J.

[CR22] Grimshaw J (2010) A guide to knowledge synthesis: a knowledge synthesis chapter. Report prepared for the Canadian Institutes of Health Research. http://www.cihr-irsc.gc.ca/e/41382.html

[CR23] Hamel LM, Robbins LB, Wilbur J (2011). Computer- and web-based interventions to increase preadolescent and adolescent physical activity: a systematic review. J Adv Nurs.

[CR24] Harvey AE, Lashinger LM, Hursting SD (2011). The growing challenge of obesity and cancer: an inflammatory issue. Ann N Y Acad Sci.

[CR25] Heath GW, Brownson RC, Kruger J, Miles R, Powell KE, Ramsey LT (2006). The effectiveness of urban design and land use and transport policies and practices to increase physical activity: a systematic review. J Phys Act Health.

[CR26] Heath GW, Parra DC, Sarmiento OL (2012). Evidence-based intervention in physical activity: lessons from around the world. Lancet.

[CR27] Higgins JPT, Green S (2011) Cochrane handbook for systematic reviews of interventions Version 5.1.0 The Cochrane Collaboration. http://www.cochrane-handbook.org. Accessed 17 Feb 2016

[CR28] Hinrichs T, Brach M (2012). The general practitioner’s role in promoting physical activity to older adults: a review based on program theory. Curr Aging Sci..

[CR29] Kohl H, Craig CL Lambert EV et al (2012) The pandemic of physical inactivity: global action for public health. Lancet. doi:10.1016/S0140-6736(12)60898-8 10.1016/S0140-6736(12)60898-8 22818941

[CR30] Kumanyika SK, Swank M, Stachecki J, Whitt-Glover MC, Brennan LK (2014). Examining the evidence for policy and environmental strategies to prevent childhood obesity in black communities: new directions and next steps. Obes Rev.

[CR31] Lee YH, Pratley RE (2005). The evolving role of inflammation in obesity and the metabolic syndrome. Curr Diab Rep.

[CR32] Levac D, Colquhoun H, O’Brien KK (2010). Scoping studies: advancing the methodology. Implement Sci.

[CR33] Levy Y, Ellis TJ (2006). A systems approach to conduct an effective literature review in support of information systems research. Informing Science.

[CR34] Lin V, Jones CM, Synnot A, Wismar M (2012) Chapter 2: Synthesizing the evidence:how governance structures can trigger governance actions to support Health in All policies. In: McQueen DV, Wismar, M, Lin V, Jones CM, Davies M (eds) Intersectoral Governance for Health in All policies: structures, actions and experiences European Observatory on Health Systems and Policies Observatory Studies Series No. 26

[CR35] Martin BW, Kahlmeier S, Racioppi F, Berggren F, Miettinen M, Oppert JM (2006). Evidence-based physical activity promotion—HEPA Europe, the European Network for the Promotion of Health-Enhancing Physical Activity. J Public Health.

[CR36] Martin-Diener E, Kahlmeier S, Vuillemin A, van Mechelen W, Vasankari T, Racioppi F, Mart BW (2014). 10 years of HEPA Europe: what made it possible and what is the way into the future?. Scweizerische Z Sportmed Sporttraumatol.

[CR37] Matson-Koffman DM, Brownstein JN, Neiner JA, Greaney ML (2005). A site specific literature review of policy and environmental interventions that promote physical activity and nutrition for cardiovascular health: what works?. Am J Health Promot.

[CR38] McPherson AC, Keith R, Swift JA (2014). Obesity prevention for children with physical disabilities: a scoping review of physical activity and nutrition interventions. Disabil Rehabil.

[CR39] Ndumbe-Eyoh S, Moffatt H (2013). Intersectoral action for health equity: a rapid systematic review. BMC Public Health.

[CR40] Packer N, Hoffman-Goetz L, Ward G (2010). Does physical activity affect quality of life, disease symptoms and immune measures in patients with inflammatory bowel disease? a systematic review. J Sports Med Phys Fit.

[CR41] Pate RR, Pratt M, Blair SN (1995). Physical activity and public health. A recommendation from the Centers for Disease Control and Prevention and the American College of Sports Medicine. JAMA.

[CR42] Patrick K, Pratt M, Sallis RE (2009). The healthcare sector’s role in the US national physical activity plan. J Phys Act Health.

[CR43] Rantala R, Bortz M, Armada F (2014). Intersectoral action: local governments promoting health. Health Promot Int.

[CR44] Romeo J, Wärnberg J, Pozo T, Marcos A (2010). Physical activity, immunity and infection. Proc Nutr Soc.

[CR45] Rütten A, Abu-Omar K, Gelius P, Frahsa A, McQueen DV (2013). Physical inactivity and health promotion: evidence and challenges. Global handbook on noncommunicable diseases and health promotion.

[CR46] Rychetnik L, Frommer M, Hawe P, Shiell A (2002). Criteria for evaluating evidence on public health interventions. J Epidemiol Community Health.

[CR47] Sallis JF, Bauman A, Pratt M (1998). Environmental and policy interventions to promote physical activity. Am J Prev Med.

[CR48] Sallis JF, Cervero RB, Ascher W, Henderson KA, Kraft MK, Kerr J (2006). An ecological approach to creating active living communities. Annu Rev Public Health.

[CR49] Schmid TL, Pratt M, Witmer L (2006). A framework for physical activity policy research. J Phys Act Health.

[CR50] Shankardass K, Solar O, Murphy K, Greaves L, O’Campo P (2012). A scoping review of intersectoral action for health equity involving governments. Int J Public Health.

[CR51] Shill J, Mavoa H, Crammond B, Loff B, Peeters A, Lawrence M, Allender S, Sacks G, Swinburn BA (2012). regulation to create environments conducive to physical activity: understanding the barriers and facilitators at the Australian state government level. PLoS ONE.

[CR52] Council of the European Union is as follows: The Council of the European Union (2013). Legislative acts and Other Instruments. Council recommendation on promoting health-enhancing physical activity across sectors. Brussels. http://ec.europa.eu/sport/library/documents/hepa_en.pdf

[CR53] United States Department of Health and Human Services (1996) Physical activity and health: a report of the Surgeon General. US Department of Health and Human Services, Public Health Service, CDC, National Center for Chronic Disease Prevention and Health Promotion, Atlanta, GA

[CR54] United States Department of Health and Human Services (2008) Physical activity guidelines advisory committee: Physical activity guidelines advisory committee report US. DHHS, Washington, DC

[CR55] Vancampfort D, De Hert M, Skjerven LH, Gyllensten AL, Parker A, Mulders N, Nyboe L, Spencer F, Probst M (2012). International Organization of Physical Therapy in Mental Health consensus on physical activity within multidisciplinary rehabilitation programmes for minimising cardio-metabolic risk in patients with schizophrenia. Disabil Rehabil.

[CR56] Vestmark N, Kahlmeier S, Racioppi F (2011). Promoting sport and enhancing health in European Union countries: a policy content analysis to support action.

[CR57] Wiseman MJ (2010). Deriving policy from evidence: experience from the WCRF/AICR report. Crit Rev Food Sci Nutr.

[CR59] World Health Organization (2004). Global strategy on diet, physical activity and health.

[CR60] World Health Organization (2009). Interventions on diet and physical activity: What works: summary report.

[CR61] World Health Organization (2014). First meeting of the European Union Physical Activity Focal Points Network Rome, Italy 21–22 October 2014.

