# Supplementary material for: Three types of scientific evidence to inform physical activity policy: results from a comparative scoping review
Source: Int J Public Health. 2016 Apr 26;61:553–63. doi: 10.1007/s00038-016-0807-y (PMC4947117; doi:10.1007/s00038-016-0807-y)
Supplement: Supplementary file 1 — Supplementary material 1 (PDF 175 kb) [file 38_2016_807_MOESM1_ESM.pdf]

International Journal of Public Health (IJPH)

Three types of scientific evidence to inform physical activity policy: Results from a comparative scoping review

Alfred Rütten,[1] Diana Schow, João Breda, Gauden Galea, Sonja Kahlmeier, Jean-Michel Oppert, Hidde van der Ploeg, Willem van Mechelen

Additional File 1: Supplementary tables and figures

Additional Table 1 *Kinds of reviews identified per each type of evidence: type I health, type II intervention, type III policy*

	**Type I**		**Type II**		**Type III**	
**Review Types**	**Number**	**%**	**Number**	**%**	**Number**	**%**
Review (not systematic)	451	52.7	108	30.9	24	60
Systematic Review	266	31.1	157	44.8	6	15
Meta Analysis	68	7.9	42	12.0	2	5
Review of Review	18	2.1	21	6.0	2	5
Narrative Review	11	1.3	4	1.1	0	.0
Synthesis – Integrative Review	8	0.9	11	3.1	1	2.5
Scoping Review	5	0.6	0	.0	2	5.0
Document Review	5	0.6	0	.0	0	.0
Bibliography Review	3	0.4	0	.0	0	.0
Rapid Review	2	0.2	0	.0	0	.0
Umbrella Review	1	0.1	0	.0	0	.0
Qualitative Review	1	0.1	0	.0	0	.0
Other	17	2.0	7	2.0	3	7.5

As shown in Additional Table 1, systematic reviews and meta-analyses are most prominent regarding Type II evidence (more than 50%) and represent almost 40% of selected reviews of Type I evidence. In contrast, these approaches only represent 20% of the Type III reviews. Review types were marked "other" if the title and abstract did not provide sufficient information to determine the category.

[1] Corresponding author: Alfred Rütten, Friedrich Alexander University, Institute of Sport Science and Sport, Erlangen, Germany; e-mail: alfred.ruetten@fau.de; phone: +49 (0) 9131.85.25.000; fax +49 (0) 9131.85.25.002

Additional Figure 1 Classifications of type II (intervention) reviews based on settings

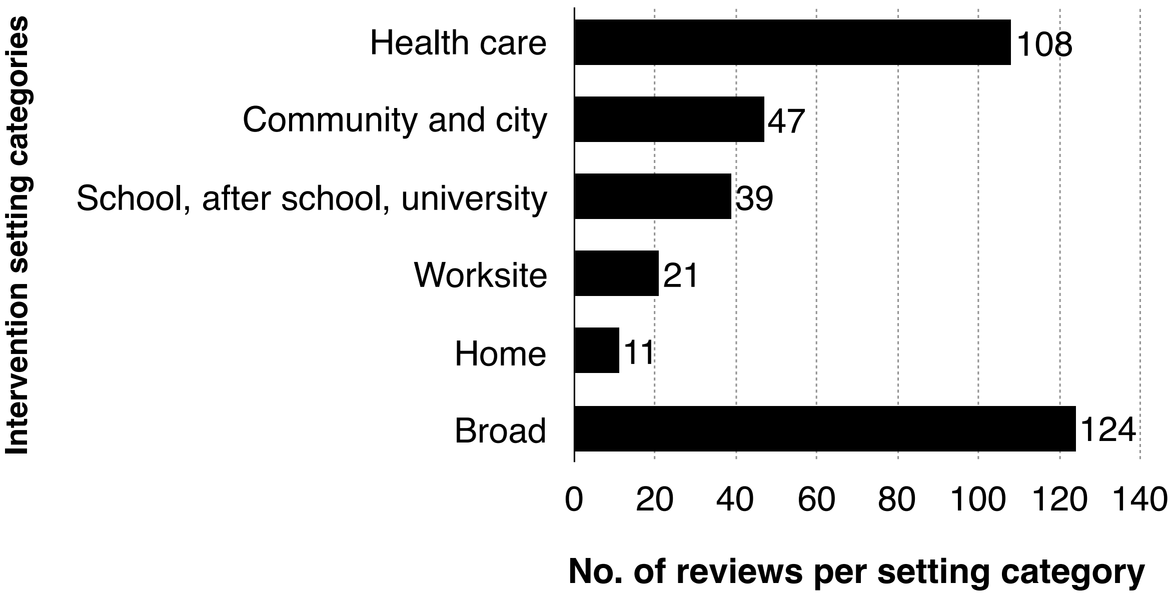

As shown in Additional Figure 1, the most prevalent setting in Type II was health care (31%). This was followed by community and city (13%), school, after school (i.e. the after school period during the day), university (11%), worksite (6%) and home (3%), respectively. Thirty-five percent of reviews were categorized as broad if they addressed multiple settings or were not focused on settings.

Additional Figure 2 *Classifications of type II (intervention) reviews based on intervention types*

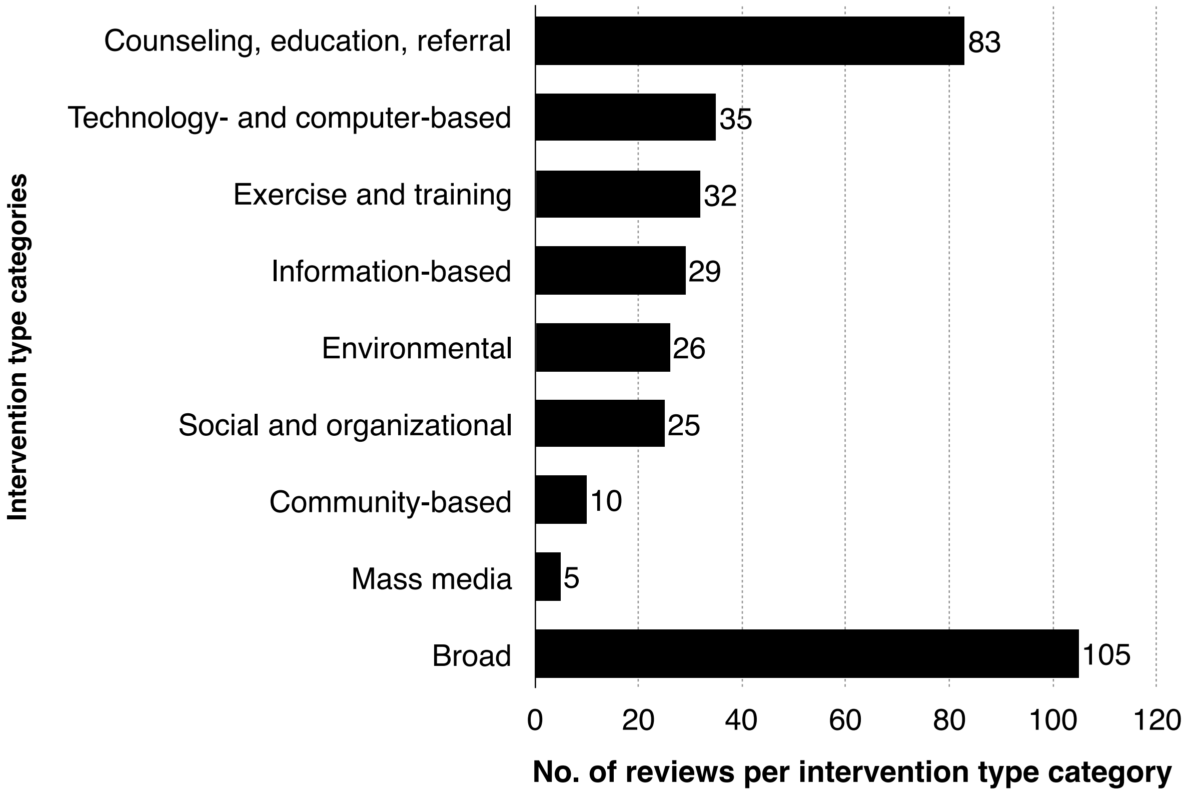

As illustrated in Additional Figure 2, the most prevalent intervention type in Type II reviews was counseling/education/referral (24%). This was followed by technology and computer-based (10%), exercise and training (9%), information-based (8%), enironmental (7%), social and organizational (7%), community-based (3%) and mass media (1%). Thirty percent of reviews were categorized as "broad" if they addressed multiple intervention types or were not focused on interventions.[2]

[2] Percentages do not add up due rounding issues.
